# Longitudinal and Transverse ^1^H Nuclear Magnetic Resonance Relaxivities of Lanthanide Ions in Aqueous Solution up to 1.4 GHz/33 T

**DOI:** 10.3390/molecules29204956

**Published:** 2024-10-19

**Authors:** Rami Nasser Din, Aiswarya Chalikunnath Venu, Thomas Rudszuck, Alicia Vallet, Adrien Favier, Annie K. Powell, Gisela Guthausen, Masooma Ibrahim, Steffen Krämer

**Affiliations:** 1 Université Grenoble Alpes, LNCMI-EMFL, CNRS, INSA-T, and UPS, CEDEX 9, 38042 Grenoble, France; rami.nasser-din@inrae.fr; 2 Institute of Nanotechnology, Karlsruhe Institute of Technology, 76131 Karlsruhe, Germany; aiswarya.venu@kit.edu (A.C.V.); annie.powell@kit.edu (A.K.P.); masooma.ibrahim@kit.edu (M.I.); 3 Institute of Mechanical Process Engineering and Mechanics, Karlsruhe Institute of Technology, 76131 Karlsruhe, Germany; trudszuck@v-er.eu (T.R.);; 4 Université Grenoble Alpes, CNRS, CEA, Institut de Biologie Structurale (IBS), 38044 Grenoble, France; alicia.vallet@ibs.fr (A.V.); adrien.favier@ibs.fr (A.F.); 5 Institute of Inorganic Chemistry, Karlsruhe Institute of Technology, 76131 Karlsruhe, Germany; 6 Institute for Quantum Materials and Technologies, Karlsruhe Institute of Technology, 76131 Karlsruhe, Germany; 7 Chair of Water Chemistry and Water Technology, Engler-Bunte-Institut, Karlsruhe Institute of Technology, 76131 Karlsruhe, Germany

**Keywords:** nuclear magnetic resonance relaxation dispersion, paramagnetic relaxation enhancement, lanthanide ions, ultra-high magnetic fields, magnetic field homogeneity, magnetic field stability

## Abstract

The longitudinal and transverse nuclear magnetic resonance relaxivity dispersion (NMRD) of ^1^H in water induced by the paramagnetic relaxation enhancement (PRE) of dissolved lanthanide ions (Ln^3+^) can become very strong. Longitudinal and transverse ^1^H NMRD for Gd^3+^, Dy^3+^, Er^3+^ and Ho^3+^ were measured from 20 MHz/0.47 T to 1382 MHz/32.5 T, which extended previous studies by a factor of more than two in the frequency range. For the NMRD above 800 MHz, we used a resistive magnet, which exhibits reduced field homogeneity and stability in comparison to superconducting and permanent NMR magnets. These drawbacks were addressed by dedicated NMRD methods. In a comparison of NMRD measurements between 800 MHz and 950 MHz performed in both superconducting and resistive magnets, it was found that the longitudinal relaxivities were almost identical. However, the magnetic field fluctuations of the resistive magnet strongly perturbed the transverse relaxation. The longitudinal NMRDs are consistent with previous work up to 600 MHz. The transverse NMRD nearly scales with the longitudinal one with a factor close to one. The data can be interpreted within a PRE model that comprises the dipolar hyperfine interactions between the ^1^H and the paramagnetic ions, as well as a Curie spin contribution that is dominant at high magnetic fields for Dy^3+^, Er^3+^ and Ho^3+^. Our findings provide a solid methodological basis and valuable quantitative insights for future high-frequency NMRD studies, enhancing the measurement accuracy and applicability of PRE models for paramagnetic ions in aqueous solutions.

## 1. Introduction

The investigation of paramagnetic relaxation enhancement (PRE) has been a subject of enduring interest since the early days of nuclear magnetic resonance (NMR) [1]. PRE is generated by the fluctuating hyperfine interaction between the magnetic moment of the paramagnetic (PM) compound in solution and the nuclear spin of the environment, often ^1^H of water. This process enhances the longitudinal ($R_{1}$) and transverse ($R_{2}$) nuclear spin relaxation rates. The PRE efficiency of a PM compound is quantitatively measured by its NMR relaxivities $r_{i}$ (i=1,2), which are defined by the longitudinal ($R_{1}$) and transverse ($R_{2}$) NMR relaxation rates divided by the concentration *c* of the compound. $r_{i}$ for a PM ion or molecule depend on the size of its magnetic moment and its electronic relaxation times, as well as on chemical exchange and rotational and diffusion processes in a given solution. In high magnetic fields, a Curie spin contribution can occur, which originates from the interaction of the nuclear spin with the time-averaged magnetic moment of the PM compound [2,3]. Standard models distinguish between inner sphere relaxation originating from solvent molecules in the first coordination sphere of the PRE compound, described by the Solomon–Bloembergen–Morgan theory [4,5,6], and outer sphere relaxation originating from interactions between the PM compound with non-coordinated molecules of the solvent, described by the Hwang–Freed theory [7,8]. Comprehensive reviews on PRE can be found in [9,10,11].

Although PRE generated by aqueous solutions of lanthanide ions (Ln^3+^) with a total spin J>0 has been studied for many decades [12,13,14,15,16,17], it is still of interest [18]. Ln^3+^ exhibit short electronic relaxation times, $\tau_{s}\approx 10^{-13}$ s, except for Gd^3+^, where $\tau_{s}\approx 10^{-8}$–$10^{-10}$ s [11]. This allows the Curie spin contribution to be observed at high frequencies, as shown in previous NMRD studies on Dy^3+^, Ho^3+^, and Er^3+^ up to 600 MHz [19]. For frequencies below 100 MHz, their relaxivities are almost field-independent due to a dominant dipolar contribution modulated by the ions’ short electronic relaxation times. The NMRD of Gd^3+^ exhibits a dispersion step between 1 and 20 MHz, followed by a decrease towards a plateau [14].

Most ^1^H NMRD studies of PRE end up at frequencies below 800 MHz/18.8 T [20]. However, recent progress in superconducting (SC) magnets has opened the way for a new generation of very homogeneous and stable high-field magnets. Nowadays, commercial high-resolution NMR magnets operate up to 1200 MHz/28.2 T [21]. Despite these achievements, water-cooled, high-electrical-power resistive magnets at dedicated high magnetic field facilities like the LNCMI Grenoble [22] still exceed these field strengths and enable NMRD at higher magnetic fields up to 1.4 GHz/33 T, as first shown by studies of ${\mathrm{Fe}}_{10}$${\mathrm{Dy}}_{10}$ clusters [23] and, more recently, for studies of paramagnetic polyoxometalate (PM-POM) compounds [24,25]. However, due to their limitations in field homogeneity and stability, NMR studies of such magnets require tailored methods in order to overcome these drawbacks [26,27].

In this context, our study of water ^1^H NMRD over a wide range of frequencies from 20 to 1382 MHz for aqueous solutions of Ln^3+^ ions with Ln^3+^∈$\left\{{\mathrm{Gd}}^{3+},\;{\mathrm{Dy}}^{3+},\;{\mathrm{Ho}}^{3+},\;{\mathrm{Er}}^{3+}\right\}$ had two objectives: an investigation of the possible impacts of the resistive field environment on NMR relaxation experiments and the extension of previous ^1^H NMRD studies of water with dissolved Ln^3+^ up to frequencies more than two times higher.

Although resistive magnets have regularly been used in the last ten years for NMRD studies of various compounds [23,24,25], the impact of their limitations has not been quantitatively investigated. Due to the well-understood NMRD of Ln^3+^ ions in aqueous solutions, they are very suitable candidates to fill this knowledge gap. For this purpose, we compared their relaxivities, measured in two state-of-the-art SC magnets at 800 and 950 MHz, with those in a resistive magnet operating in the same frequency range. This approach made it possible to validate our experimental methods for NMRD in resistive magnets, as well as to identify and investigate the systematic errors caused by their limited field homogeneity and stability. This is very important for future NMRD studies on more complex compounds at such high magnetic fields.

In addition to the methodological aspect of our study, the NMRD of aqueous Ln^3+^ solutions up to 1382 MHz is also of interest for fine-tuning the existing PRE parameters, since no experimental data were available above 600 MHz. Our objective was to explore whether the observed NMRD plateau for Gd^3+^ persists at higher fields [18] and how the Curie spin contribution evolves for Dy^3+^, Ho^3+^, and Er^3+^. Using a simulation with an inner sphere relaxation model based on the Solomon–Bloembergen–Morgan theory, an outer sphere model based on Hwang–Freed theory, and a Curie spin contribution, we found electronic and rotational correlation times for Dy^3+^, Ho^3+^ and Er^3+^ that are slightly shorter than previously reported [19].

## 2. Results and Discussion

### 2.1. Comparative PRE Studies in Superconducting and Resistive Magnets Above 800 MHz

**Figure 1:**
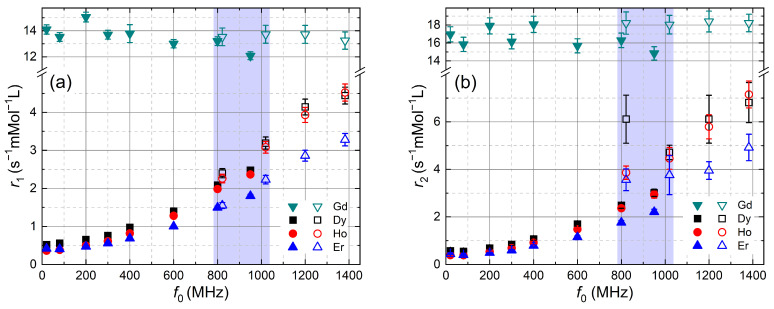
^1^H NMRD of (**a**) $r_{1}$ and (**b**) $r_{2}$ of water with dissolved LnCl_3_ salts, with Ln ∈ {Gd, Dy, Ho, Er} for Larmor frequencies $f_{0}$ from 20 MHz to 1382 MHz and at 298 K. Closed symbols are $r_{i}$ measured in permanent (20 MHz and 80 MHz) and SC magnets, and open symbols are data from the resistive magnet. The blue region marks the zone that allows for a comparison of results from SC and resistive magnets. $r_{1}$ values measured in the resistive magnet are slightly larger, which we attribute to a difference in temperature. $r_{2}$ almost scale with $r_{1}$ except in the blue region. There, $r_{2}$ values of the resistive magnet are strongly affected by field fluctuations.

Figure 1 shows the experimental ^1^H NMRD of $r_{1}$ and $r_{2}$ from 20 MHz to 1382 MHz for the studied aqueous Ln^3+^ ion solutions with Ln^3+^∈ {Gd^3+^, Dy^3+^, Ho^3+^, Er^3+^}. The temperature was 298 ± 2 K. Note that a linear scale is used for all axes. $r_{1}$ are consistent with previously reported data [14,18,19,28]. The NMRD of $r_{1}$ for Dy^3+^, Ho^3+^, and Er^3+^ increases monotonically with the field due to the Curie contribution, whereas it is roughly field independent for Gd^3+^.

The blue shaded regions mark the zone that allows for a comparison between $r_{i}$ measured in state-of-the-art SC magnets (800 MHz and 950 MHz) and the resistive magnet (822 MHz and 1020 MHz, open symbols). For the following comparison, we use the data of the SC magnets as a reference, since they exhibit smaller error bars. In a first approximation, there is no big difference in the longitudinal relaxivities $r_{1}$. However, a detailed analysis shows that $r_{1}$ values obtained in the resistive magnet are systematically larger. This is visible as a small step at comparable fields (800 and 950 MHz). By interpolating $r_{1}$ for Dy^3+^, Ho^3+^, and Er^3+^, one can calculate the relative step size, $\left(r_{1,\mathrm{res}}-r_{1,\mathrm{SC}}\right)/r_{1,\mathrm{SC}}$ of $r_{1,\mathrm{res}}$ of the resistive magnet with respect to $r_{1,\mathrm{SC}}$ of the superconducting magnets, which are taken as a reference. This step size is 5% at 800 MHz and 12% at 950 MHz. This behavior could be explained by an increase of $r_{1}$ in the resistive magnet originating from very fast field fluctuations at $f_{0}$ that generate an additional spectral density contribution or by a temperature difference. We rule out the first cause with high probability, since the inductances of the magnets extremely weaken their field fluctuations at these high frequencies.

In order to further explore a possible temperature effect, we studied the temperature dependence of $R_{1}$ for *c* = 10 mMol $L^{-1}$ at 950 MHz in the range from 293 K to 304 K using a precise, previously calibrated temperature regulation setup for this SC magnet. Since the $R_{1}\left(c\right)$ was found to be almost perfectly linear, the temperature dependence of $R_{1}$(*c* = 10 mMol $L^{-1}$) directly provides the temperature dependence of $r_{1}$. For all Ln^3+^, we found a decreasing $r_{1}$ with temperature, which is consistent with previous results [19]. A quantitative analysis for the relative slope of the temperature dependence of $r_{1}$ at 298 K, $\left(r_{1}\left(T\right)-r_{1}\left(298\;K\right)\right)/(\left(T-298\;K\right)r_{1}\left(\left(298\;K\right)\right)$ gives values from −2.2 to −2.4 %/K. In order to explain the observed differences of $r_{1}$, the temperature of the sample in the resistive magnet must have been 2–5 K lower than in the SC magnet. In order to improve the quality of PRE studies in resistive magnets, we need to further explore this issue and develop better temperature monitoring and regulation setups.

The ^1^H NMRD of $r_{2}$ is shown in Figure 1b. For high magnetic fields, we were only able to measure $R_{2}$ data of water with dissolved Ln^3+^ for *c* = 10 mMol $L^{-1}$. For lower concentrations, the experiments did not provide the expected mono-exponential decays. The $r_{2}$ values are therefore the values of $R_{2}$ normalized by *c* = 10 mMol $L^{-1}$, assuming the linear dependence, which was always observed in SC magnets.

**Figure 2:**
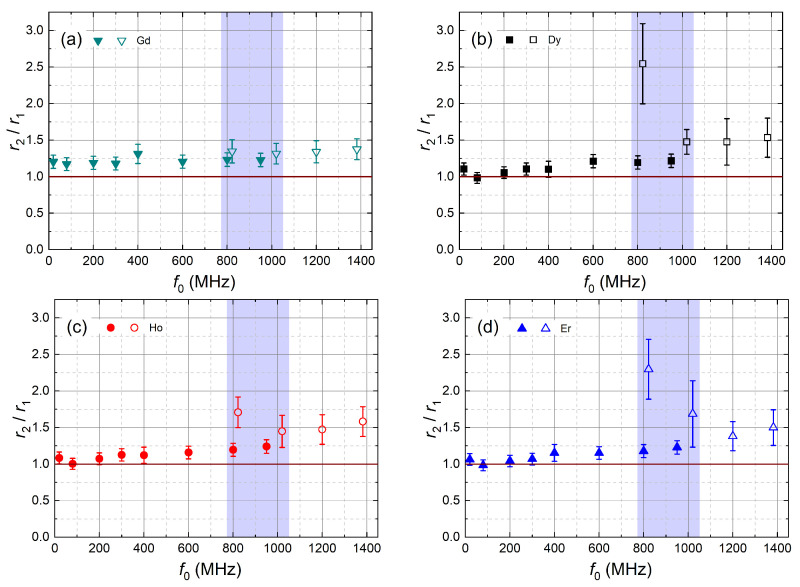
Frequency dependence of the ratio between transverse ($r_{2}$) and longitudinal ($r_{1}$) relaxivities for (**a**) Gd^3+^, (**b**) Dy^3+^, (**c**) Ho^3+^ and (**d**) Er^3+^. Closed symbols are derived from $r_{i}$ measured in permanent (20 MHz and 80 MHz) and SC magnets, and open symbols are data from the resistive magnet. $r_{2}/r_{1}$ is close to one for low frequencies, and it increases slightly with frequency for Dy, Ho and Er. In the blue zone, the ratios obtained in the resistive magnet (open symbols) strongly deviate from the general trend except for Gd^3+^ (**a**). The effect is very strong at 822 MHz and still visible at 1020 MHz (open symbols). Above 1200 MHz, the ratio for all Ln^3+^ values again follows the common trend. This behavior is explained by field fluctuations of the resistive magnet. They induced systematic errors in transverse relaxation experiments at small $R_{2}$.

For frequencies below 600 MHz and above 1200 MHz, $r_{2}$ and $r_{1}$ exhibit a similar frequency dependence for all Ln^3+^ (Figure 1). Therefore, we calculated the $r_{2}/r_{1}$ ratio (Figure 2). $r_{2}/r_{1}$ is found to be close to one and smoothly increasing with frequency, except in the region where data from both magnet types overlap. Here, the $r_{2}/r_{1}$ ratio remains smooth only for Gd^3+^, irrespective of the magnet type. For Dy^3+^, Ho^3+^ and Er^3+^, $r_{2}$ is smooth only for SC magnets, whereas the $r_{2}$ values in the resistive magnet are more than 2 times larger in this range.

As shown in the section “Materials and Methods”, we attribute this deviation to fluctuations of the resistive magnet that affect the Carr-Purcell-Meiboom–Gill (CPMG) pulse sequence used for the $R_{2}$ experiment [29,30]. If the duration of the CPMG sequence, $t_{\mathrm{CPMG}}=5\;R_{2}^{-1}$, becomes longer than the correlation time of the field fluctuations, $\tau_{\mathrm{ext}}$, the refocusing π pulses in the sequence become ineffective due to off-resonant effects [31]. Consequently, the magnetization decay will deviate from the mono-exponential behavior. This means that field fluctuations only affect $R_{2}$ experiments below a limit, $R_{2,\min}$, that depends on $\tau_{\mathrm{ext}}^{-1}$. Our results show that perturbations of $r_{2}$ become less visible above 1200 MHz, e.g., for $r_{2}>$ 6 $s^{-1}$${\mathrm{mMol}}^{-1}$L. Taking into account that the $r_{2}$ values were only extracted from 10 mMol $L^{-1}$ samples, $R_{2,\min}$ equals 60 $s^{-1}$ for the LNCMI magnet. This result allows a rough estimation of $\tau_{\mathrm{ext}}$ that affects our PRE studies. The absence of perturbations during $t_{\mathrm{CPMG}}$ implies that $\tau_{\mathrm{ext}}>5\;R_{2,\min}^{-1}$, which provides $\tau_{\mathrm{ext}}>$ 83 ms.

### 2.2. Interpretation and Modeling of NMRD in Aqueous Solutions of Ln^3+^ Ions up to 1.4 GHz

**Figure 3:**
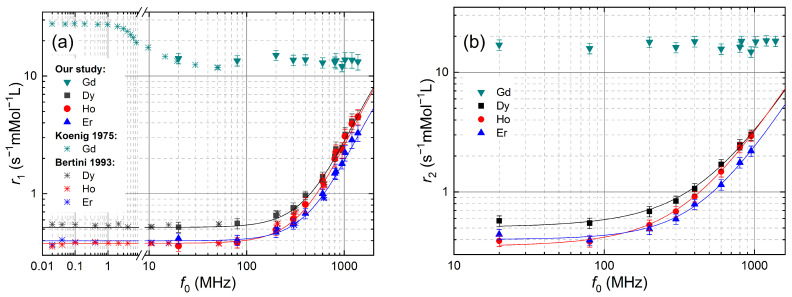
^1^H NMRD of (**a**) $r_{1}$, and (**b**) $r_{2}$, of water with dissolved Ln^3+^ ions with Ln ∈ {Gd, Dy, Ho, Er} as a function of the Larmor frequency $f_{0}$. The star symbols represent previous PRE studies performed on Ln^3+^ ions taken from the references [14,19]. The solid lines represent the theory, including the inner and outer spheres’ contributions. The parameters of the fits are summarized in Table 1.

Figure 3 depicts our ^1^H NMRD of $r_{1,2}$ for aqueous solutions of Ln^3+^ ions together with previous studies of $r_{1}$ for Dy^3+^, Ho^3+^ and Er^3+^ up to 600 MHz [19]. For better visibility, a logarithmic scale for both axes was used. Our $r_{1}$ data for Dy^3+^, Ho^3+^, and Er^3+^ are fully consistent with the previous NMRD studies. The NMRD of $r_{1}$ for Gd^3+^ exhibits a plateau, which is in the range of previously reported data [14,28].

In the well-established interpretations [14,18,19,28] for ^1^H NMRD of $r_{1}$ of Ln^3+^ ions in aqueous solutions, the inner sphere contribution dominates, whereas the outer sphere contribution is estimated to be 10%. For these compounds, the fast chemical exchange condition, $\tau_{M}\ll T_{1,\mathrm{dip}}$, is valid [32]. The difference between Gd^3+^ on the one hand and Dy^3+^, Ho^3+^, and Er^3+^ on the other hand is explained by the electronic relaxation time $\tau_{S}$ of the former being 3-5 orders of magnitude longer, $\tau_{S}$ ≈ $10^{-8}$–$10^{-10}$ s, compared to the latter, $\tau_{S}$ ≈ $10^{-13}$ s.

### 2.3. The Case of Gd^3+^

For Gd^3+^, the NMRD is explained by a dominant contribution of a rotational mechanism and a total correlation time $\tau_{c}$ = 5 × $10^{-11}$ s. The form of the NMRD at high fields requires the introduction of a frequency-dependent electron relaxation time $T_{1,e}$. It was reported that the rotational correlation time, $\tau_{R}$, and the electron correlation time, $\tau_{S}$, are of the same order of magnitude above 10 MHz [11,16,33]. Furthermore, it was shown that the Curie spin contribution is negligible [34,35]. Since our NMRD profile of $r_{1}$ for Gd^3+^ remains constant from 20 to 1382 MHz and no decrease is observed at high fields, we conclude that the condition $\omega_{I}^{2}\tau_{c}^{2}\ll 1$ is still valid up to $f_{0}$ = 1382 MHz.

### 2.4. The Case of Dy^3+^, Ho^3+^ and Er^3+^

The NMRD of $r_{1}$ for these ions is constant below $f_{0}$ = 200 MHz, indicating that the dipolar contribution is dominating in this frequency range. Above that frequency, a sizeable NMRD due to a Curie contribution becomes visible, which increases approximately quadratically with $f_{0}$, as long as $\omega_{I}^{2}\tau_{cs}^{2}\ll 1$, where $\omega_{I}=2\pi f_{0}$ and $\tau_{cs}$ is the Curie spin correlation time as defined in the annex. The NMRD of $r_{1}$ for Dy^3+^ and Er^3+^, which have both a half-integer total angular momentum *J* = 15/2 but different effective magnetic moments, $\mu_{\mathrm{eff}}$, scale with each other, whereas for Ho^3+^ with an integer *J* = 8, the NMRD of $r_{1}$ exhibits a stronger dispersion above 80 MHz.

In a further step, we modeled the NMRD of $r_{1}$ and $r_{2}$ for Dy^3+^, Ho^3+^, and Er^3+^ using inner-sphere and outer-sphere relaxation equations based on the Solomon–Bloembergen–Morgan [4,5,6] and Hwang–Freed theories [7,8]. The equations were taken from references [3,11,20,36] and are given in the Appendix D. The modeling routine was implemented as a Matlab script, with initial parameters from [19]. We fixed the magnetic moments of the Ln^3+^ ions (Table A3), the number of coordinated water molecules to *q* = 8 and the average inner sphere radius to $r_{is}$ = 3.1 × $10^{-10}$ m, since $r_{is}$ was found to be constant at this value within a 1% window for Dy^3+^, Ho^3+^ and Er^3+^ [19]. In the absence of experimental data for $r_{os}$ for Dy^3+^, Ho^3+^ and Er^3+^, we chose a constant closest outer sphere distance $r_{os}$ = 4.2 × $10^{-10}$ m using the value for Gd^3+^ found in [14]. The temperature was set to *T* = 298 K. We used the values from [32] for the chemical exchange time $\tau_{M}$, and we took the relative diffusion constant of pure water at 298 K, $D=2.62\times 10^{-9}$ m^2^$s^{-1}$ for the diffusion process. Two parameters, $\tau_{S}$ and $\tau_{R}$, were extracted from the fitting and are listed in Table 1. The extracted values of $\tau_{S}$ and $\tau_{R}$ are slightly shorter than those reported in [19]. This difference could be due to the inclusion of the outer sphere relaxation into the fit model of the NMRD, which accounts for about 10% of the total relaxivity. However, this requires further investigations, including a rigorous analysis of all systematic and statistical errors of our study.

For $r_{2}$ modeling, we only used the data obtained in SC magnets due to the previously discussed problems of $r_{2}$ in the resistive magnet. The NMRD of $r_{2}$ almost scales with $r_{1}$ with a factor close to one that slightly increases with $f_{0}$ (Figure 2). This was explained by the absence of a contact term in the relaxation rates [14,19]. Our model for $r_{2}$ uses the expressions from ref. [20]. Using the same parameters as for the $r_{1}$ modeling, assuming an absence of contact shift and fixing $\tau_{1S}=\tau_{2S}$, we obtain the curves in Figure 3b.

## 3. Materials and Methods

### 3.1. PRE Studies in Resistive Magnets

PRE studies involve longitudinal and transverse NMR relaxation experiments. The corresponding NMR sequences are well established. Inversion recovery and saturation recovery sequences provide the longitudinal relaxation rates $R_{1}$ [37,38]. The transverse relaxation rates $R_{2}$ are obtained from CPMG experiments [29,30]. Each pulse sequence comprises excitation, the evolution of variable duration ($t_{\mathrm{var}}$), and detection. Between two subsequent pulse sequences, a recovery delay is inserted (up to $5\;R_{1}^{-1}$). During the excitation part, the nuclear magnetization ***M*** is driven out of its equilibrium state $M_{\mathrm{eq}}=M_{\mathrm{eq}}e_{z}$, assuming the external magnetic field $B_{0}$ along the *z*-axis. For this purpose, one or several radio-frequency (rf) pulses are used at the Larmor frequency $f_{0}=\gamma_{n}B_{0}$, where $\gamma_{n}$ is the gyromagnetic ratio of the nucleus. Their spectral excitation width is related to the nutation frequency $f_{1}$ that is proportional to the strength of the rf field, $B_{1}$, generated by an rf power amplifier. The evolution during $t_{\mathrm{var}}$ can contain additional pulses, like for the CPMG sequence. The fluctuating hyperfine interactions between the PM compound and the nucleus in the solution molecule generate the nuclear spin relaxation during this period. The detection starts after an optional readout pulse. During that period, $M(t_{\mathrm{var}})$ is recorded in a rotating frame precessing along $B_{0}$ with $f_{0}$. $M_{\mathrm{eq}}$ is finally reestablished during the recovery delay.

Field inhomogeneities create Larmor frequency variations in the space $\Delta f\left(r\right)=f\left(r\right)-f_{0}$ over typical sample size volumes of 1 cm^3^. Temporal field variations generate deviations from $f_{0}$ over the entire sample volume $\Delta f\left(t\right)=f\left(t\right)-f_{0}$. Both Δf(r) and Δf(t) are typically much smaller than the nutation frequency $f_{1}$ for NMR experiments in SC magnets. This ensures homogeneous and on-resonant excitation of the magnetization in space and time during the entire pulse sequence. Typical values are $\Delta f\left(r,t\right)/f_{0}$ = $10^{-6}$ to $10^{-9}$. Moreover, Δf(t) in such magnets does not generate additional relaxation processes of ***M***.

In water-cooled, high-electrical-power resistive magnets, however, these conditions are not found. Their field inhomogeneities and temporal field variations are 100–10,000 times larger than in state-of-the-art superconduction magnets. Moreover, two different categories of temporal field variations occur: (i) field drifts that are constant during the entire pulse sequence (typically 1 s) and (ii) field fluctuations that occur on shorter timescales (typically 20–100 ms). They are mostly due to remaining ripples of the power convertors at 50 Hz and imperfections of their regulation as well as mechanical vibrations induced by the water cooling pipes.

In the following sections, we present, analyse, and discuss our approach to overcome these problems for the case of the resistive magnet at LNCMI Grenoble. A more detailed discussion can be found in [39].

#### 3.1.1. NMR Relaxivity Experiments in Inhomogeneous Static Magnetic Fields

**Figure 4:**
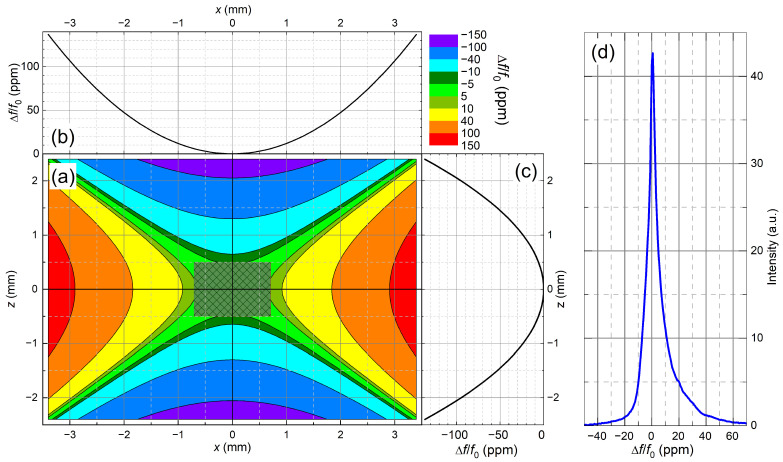
(**a**) Contour plot of the relative spatial field deviation $\Delta f\left(z,r\right)/f_{0}$ from the value in the center in the (*z*,*x*)-plane for the LNCMI M9 resistive magnet according to Equation (1). (**b**,**c**) show cuts in the radial and axial directions. The marked region at the center shows the optimum position for a sample that has the form of a horizontal cylinder of 1 mm diameter and 1.5 mm length. The line broadening amounts here to 15 ppm. (**d**) A ^1^H NMR spectrum of water recorded at 1020 MHz/24 T confirms this approach.

Figure 4 shows the calculated spatial variation of $f_{0}$ of the LNCMI M9 24 MW resistive magnet near its center. An axisymmetric model for the solenoid magnet has been used. The dominating term of the spatial field deviation, Δf(r,z), from the value in the center, $f_{0}$, in axial, *z*, and radial, *r*, directions in cylindrical coordinates is given by

(1) $$\Delta f\left(r,z\right)=f_{0}\;G_{\mathrm{zz}}\left(z^{2}-\frac{1}{2}r^{2}\right),$$

where $r=\sqrt{x^{2}+y^{2}}$ is the radial coordinate. $G_{\mathrm{zz}}$ = 1/($2f_{0}{)\;\partial^{2}f\left(r,z\right)/\partial z^{2}|}_{\left(z,r)=(0,0\right)}$ is proportional to the second-order axial gradient of f(r,z) at the center [40], which amounts to $G_{\mathrm{zz}}$ = −25 ppm/mm^2^ for the magnet used here [41]. The axial second-order gradient (Figure 4c) is two times larger than the radial one (Figure 4b). Therefore, the use of the standard 5 mm NMR sample tube volumes would cause NMR linewidths of about 160 ppm, i.e., more than 220 kHz for $f_{0}$ = 1382 MHz. This is far beyond the excitation bandwidths $f_{1}$ of our NMR probe, which are between 110 kHz at 822 MHz and 70 kHz at 1382 MHz. In order to ensure homogeneous excitation across the sample, $\Delta f\left(r,z\right)/f_{0}$ should be below 15 ppm. This can be achieved for our magnet by reducing all sample dimensions to ≈1 mm and a precise positioning of the sample at the center of the magnetic field (better than 0.1–0.2 mm). This optimal sample size and position are marked in Figure 4a. We confirmed this approach by a ^1^H NMR spectrum of water recorded at 1020 MHz/24 T. The predicted linewidth reduction to 15 ppm is achieved for a sample that has the form of a horizontal cylinder of 1 mm diameter and 1.5 mm length (Figure 4d).

#### 3.1.2. NMR Relaxivity Experiments in Time-Varying Fields

**Figure 5:**
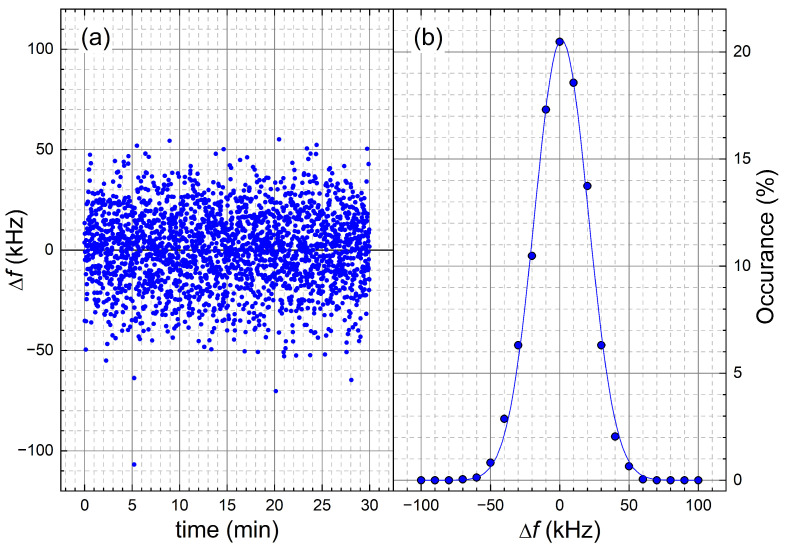
(**a**) Fluctuations Δf(t) of the LNCMI M9 resistive magnet recorded for 30 min at 1020 MHz/24 T. Δf are obtained from the spectral line positions of single-scan ^1^H NMR experiments of water with dissolved Gd^3+^ (*c* = 60 mMol $L^{-1}$). (**b**) Their distribution can be modeled by a Gaussian function with a standard deviation σ = 19 kHz.

The time variations of the magnetic field $B_{0}\left(t\right)$ generate additional variations of $f_{0}$ that are independent of the sample size. The methodological approaches to limit their influence on NMR experiments distinguish between field drifts and field fluctuations. The former can be easily corrected by measuring the value of $B_{0}$ just before the experiment and adjusting $f_{0}$ to that value. For the LNCMI-resistive magnet, the slow drifts remain below ±10 ppm during the entire experiment duration (up to 20 min. for $R_{1}$ of the pure solvent). The case of fast field fluctuations is more complicated, and all methods to limit their impact on NMR experiments require quantitative measurements of the fluctuation amplitude and time scale. The fluctuation amplitudes at 24 T/1020 MHz were recorded for 30 min by single-scan ^1^H NMR experiments of water with dissolved Gd^3+^ with *c* = 60 mMol $L^{-1}$ (Figure 5). Δf are the spectral line positions. Their distribution is Gaussian with a standard deviation σ = 19 kHz. Taking a 3σ criterion, the fluctuation range $\Delta f=\max(f_{0}\left(t\right)-\langle f_{0}\rangle$) around the mean value $\langle f_{0}\rangle$ becomes ±60 kHz. Δf(t) is independent of $f_{0}$ in the range 822–1382 MHz.

The impact of these fluctuations is different for the excitation and detection process, as well as for longitudinal and transverse relaxation experiments. After an excitation by a pulse at the radio frequency $f_{rf}=\langle f_{0}\rangle$ with a nutation frequency $f_{1}$ (Table A2) and an on-resonant pulse angle $\beta_{nom}$, the magnetization ***M*** for an off-resonant frequency Δf becomes

**Figure 6:**
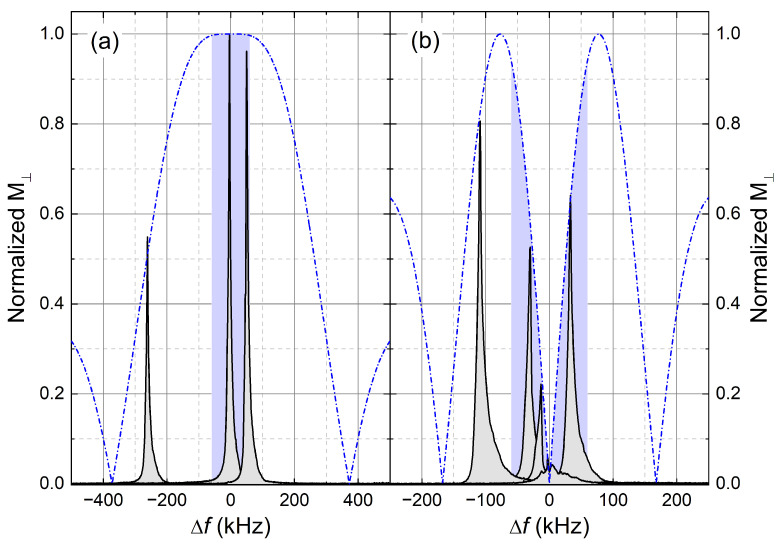
Transverse magnetization $M_{⊥}$ as a function of the field fluctuation amplitude Δf after the application of (**a**) π/2 and (**b**) π pulses at 1020 MHz/24 T. ^1^H NMR spectra of water with dissolved Gd^3+^ ions (*c* = 60 mMol $L^{-1}$) are shown. The dashed-dotted lines are theoretical curves for $M_{⊥}$ using Equation (2) for $f_{1}$ = 100 kHz. The blue area marks the range of typical field fluctuations ± 60 kHz. They strongly perturb the magnetization state after a π pulse, whereas the state after a pulse remains almost unchanged.

(2) MMeq=x1+x2cos1+x2βnom−1 11+x2sin1+x2βnom 11+x2x2+cos1+x2βnom,

where $x=\Delta f/f_{1}$ [39,42]. In Figure 6, we show the impact of fluctuations Δf(t) on the normalized transverse magnetization $M_{⊥}=\sqrt{\left(M_{x}^{2}+M_{y}^{2}\right)}/M_{eq}$ for the case of (a) $\beta_{nom}=\pi /2$ and (b) $\beta_{nom}=\pi$. The experimental spectra for the π/2 case were selected from the previously shown fluctuation experiment. The spectra for the π pulses were measured afterwards. The dashed-dotted lines are theoretical curves for $M_{⊥}$ using Equation (2) for $f_{1}$ = 100 kHz. $M_{⊥}$ after a π pulse becomes strongly perturbed by the typical field fluctuations of ±60 kHz (blue areas in Figure 6). Instead of zero, $M_{⊥}$ of up to 90% can occur; i.e., the π pulse becomes a π/2 pulse. However, $M_{⊥}$ after a pulse is more robust against fluctuations, and the deviation in intensity is less than 2%.

For $R_{1}$ experiments, the progressive saturation recovery sequence (PSR) only involves π/2 pulses [37,38] and is therefore more robust than the inversion recovery sequence (IR). This ensures a well-defined initial state $M(t_{\mathrm{rec}}=0)\;=\;0$, even under fluctuation-induced off-resonant excitation. Moreover, the PSR is less time-consuming for long relaxation times $T_{1}=R_{1}^{-1}$, since there is no waiting time of $5\;T_{1}$ after each sequence in contrast to the IR. This is more efficient for NMRD studies in resistive magnets, which are time-limited and expensive. In our work, we measured $T_{1}$ for between approximately 10 s for the solvent and approximately 6 ms for the solution of GdCl_3_ with *c* = 10 mMol $L^{-1}$.

During the detection of the $R_{1}$ experiments, fluctuations of $f_{0}$ generate frequency offset, which makes signal averaging ineffective. Therefore, single scan sequences were used for all relaxation experiments. Moreover, we developed a data processing method that enhances the signal-to-noise ratio. In addition to the standard processing methods found in commercial NMR programs, it corrects the fluctuation-induced frequency offset Δf during the detection. For each spectrum *i* of the experiment, an offset frequency $\Delta f_{i}$ is determined. All points are then shifted by $-\Delta f_{i}$ in order to obtain zero frequency offset. This facilitates phase correction and reduces the integration window size for magnetization calculation, resulting in an improvement of the signal-to-noise ratio [39].

**Figure 7:**
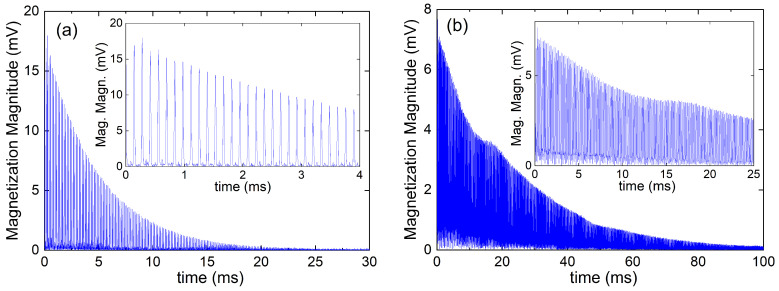
$R_{2}$ CPMG experiment of ^1^H NMR of water with dissolved (**a**) Gd^3+^ ions at 1382 MHz and (**b**) Dy^3+^ ions at 822 MHz. For both solutions, the concentration *c* was =10 mMol $L^{-1}$. The figures show the magnetization magnitude of the time records. Note the different time scales of the decays. For long $T_{2}$, the decay deviates from the expected mono-exponential behavior. This is due to off-resonant π pulses generated by the field fluctuations.

The case of $R_{2}$ experiments is more challenging. Although we applied single-scan CPMG sequences that are proven to work well in inhomogeneous and unstable magnetic fields [26], we were not able to extract reliable $R_{2}$ in the general case (Figure 7). A ^1^H CPMG sequence for a 10 mMol $L^{-1}$ Gd^3+^ solution recorded at 1382 MHz shows a mono-exponential decay (Figure 7a). The same sequence gave a non-exponential decay for a 10 mMol $L^{-1}$ Dy^3+^ solution at 822 MHz. Therefore, the $R_{2}$ obtained from a monoexponential fit no longer provides the correct transverse relaxation rate of the compound (Figure 7b). $T_{2}=1/R_{2}$ determines the duration of the sequence, typically $5\;T_{2}$, which amounts to 30 ms (Gd-case) and 100 ms (Dy-case). As soon as this duration becomes longer than the typical correlation time of the field fluctuations, $\tau_{\mathrm{ext}}$, the CPMG signal becomes strongly perturbed by off-resonance effects.

This effect has been studied in the past [31], and it depends on the π pulse duration ($t_{\pi }=1/(2\;f_{1}$)) and the duration of the interpulse delay $\Delta_{e}$ of the CPMG sequence given by ${\left(\pi /2\right)}_{y}-{\left(\Delta_{e}-\pi_{x}-\Delta_{e}\right)}_{2i}$. We provide these values for our experiments in the resistive magnet in Table A2 in the annex. We also added upper limits for $f_{\mathrm{off}}/f_{1}$ and $\Delta_{e}\;f_{\mathrm{off}}$ using our maximum off-resonance frequency $f_{\mathrm{off}}$ = 60 kHz, since they were used in [31] to quantify systematic errors induced by off-resonant effects. Our parameters are in the range where strong perturbations in the echo amplitudes are expected and can therefore explain our experimental observations. For the measurements in the superconducting magnets, $t_{\pi }$ and $\Delta_{e}$ were far away from values where systematic errors occur in CPMG sequences due to their better stability.

Apart from removing the fluctuations from the resistive magnet by an active field correction, we identify two possibilities to overcome this drawback. First, as $R_{2}$ linearly depends on the concentration *c*, an increase in *c* will lead to an increase in $R_{2}$ or a shortening of $T_{2}$ and the CPMG echo train. Therefore, $r_{2}$ can be extracted from a concentration series, where all $T_{2}\left(c\right)$ are much shorter than $\tau_{\mathrm{ext}}$. However, this workaround only works for highly soluble samples such as the investigated Ln^3+^. Second, one can use more powerful amplifiers that generate a larger $f_{1}$. This will reduce the pulse error induced by off-resonance frequencies Δf.

### 3.2. Preparation of μL-Volume Aqueous Solutions of Ln^3+^ Ions

High-purity LnCl_3_ salts with Ln ∈ {Gd,Dy,Ho,Er} were purchased from Sigma-Aldrich. We prepared a series of solutions with concentrations *c* = 10, 5, 2, 1, 0.5, and 0.2 mMol $L^{-1}$ for each LnCl_3_ compound, which gives 25 samples including the solvent. The solvent was a mixture of 90 % volume fraction D_2_O and 10% H_2_O to prevent radiation damping, which was carefully checked [39]. The solutions were stored in 5 mm Wilmad NMR sample tubes. Some portions were later transferred into 1.7 mm and 1.0 mm capillary tubes according to the requirements of the NMR instrument.

**Figure 8:**
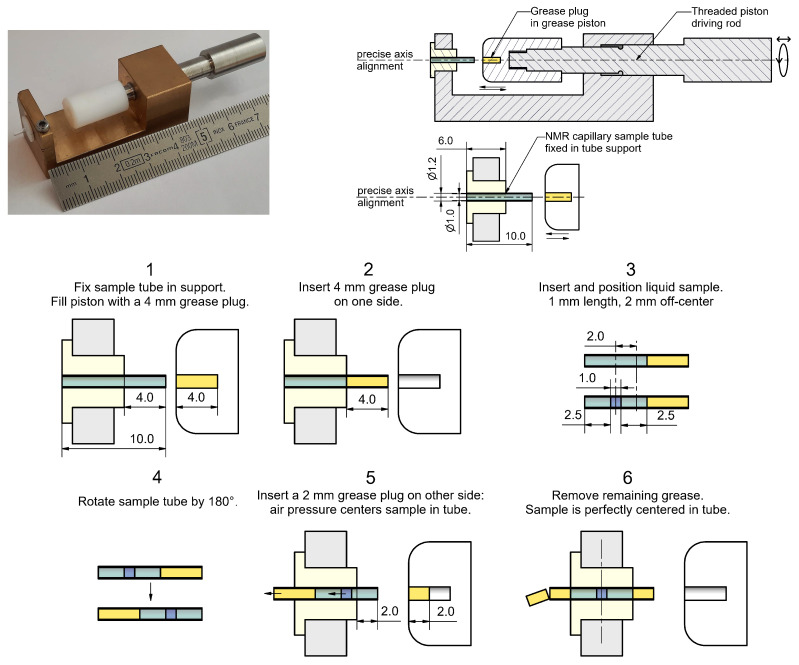
**Top left**: Sample filling tool for NMR capillary tubes of 1 mm inner diameter and 10 mm length used for the NMRD studies in the resistive magnet. **Top right**: Schematic view of the tool. A threaded driving rod for a grease piston (white) allows the insertion of small amounts of grease (yellow) into the tubes (green). The precise alignment of the piston and the tube is important to avoid the breaking of the thin walled tube. **Bottom**: Procedure for the insertion, precise positioning and sealing of 1 μL sample volumes (blue). Details are described in the text.

As described before, the NMRD studies in the resistive magnet require horizontal cylindrical samples of 1 mm diameter and 1.5 mm length. This corresponds to sample volumes of ≈1 μL. For this purpose, we decided to precisely position the sample volume in a capillary tube of 1 mm diameter and 10 mm length and close both ends with small grease plugs. We used fluorinated grease to avoid ^1^H NMR background signals. A new tool (Figure 8) was constructed for the filling process of the 25 samples to ensure perfect sample positioning and to overcome filling problems like the air pressure when sealing the tubes or the handling of small amounts of grease. The procedure of the sample filling consists of the following steps.

1. The capillary tube is fixed in the sample support. The grease piston is filled with a 4 mm grease plug.
2. The first grease plug is inserted into one end of the tube.
3. A micropipette is used to insert the 1 μL sample volume into the tube from the other end. The position is 2 mm off-center to account for the movement of the sample due to the air pressure when sealing the tube.
4. The tube is rotated by 180° and reattached to the support.
5. Two millimeters of grease are used to fill the piston and is inserted into the other end of the tube.
6. The air pressure perfectly centers the sample inside the tube. The exceeding amount of grease from the first plug can be removed.

### 3.3. NMR Instruments and Pulse Sequences

The following NMR instruments were used for our NMRD studies from 20 MHz/0.47 T to 1382 MHz/32.5 T (Table A1 in the annex):

1. Permanent magnets operating at 20 and 80 MHz at the Karlsruhe Institute of Technology (KIT), Germany.
2. Commercial Bruker SC magnets located at different facilities:
  (a) Instruments with 200, 300, and 400 MHz at KIT.
  (b) Instruments with 600 and 950 MHz at the Institut de Biologie Structurale (IBS) in Grenoble, France.
  (c) An 800 MHz magnet at the Bruker BioSpin facility in Ettlingen, Germany.
3. A resistive magnet at the Laboratoire National des Champs Magnétiques Intenses (LNCMI) in Grenoble, France, for experiments above 822 MHz.

#### 3.3.1. Bruker NMR Spectrometers up to 950 MHz

The experimental details on relaxation measurements between 20 and 400 MHz are available in reference [23]. The 600, 800, and 950 MHz spectrometers were commercial Bruker spectrometers. All these NMR spectrometers feature auto-tuning and active shimming. Data were acquired using Bruker’s TOPSPIN VERSION 3 or 4. The phase-corrected spectra were stored in 2D data files. $R_{1}$ was measured by multi-scan inversion recovery pulse sequence (IR). $R_{2}$ was obtained by a multi-scan 2D CPMG echo sequence, except for the 20 MHz, where $T_{2}$ was obtained by 1D CPMG. We used the Bruker Dynamic Center software (V.2.2) for the extraction of the $R_{1}$ and $R_{2}$ values, except for the 600 and 950 MHz spectrometers, where a Python (V.3.13.0)-based data analysis software library was developed by the IBS NMR team.

#### 3.3.2. NMR Instruments Above 820 MHz at the LNCMI Resistive Magnet

High-field NMR relaxivity experiments were performed at the Laboratoire National des Champs Magnétiques Intenses (LNCMI) in Grenoble using a 24 MW resistive magnet providing variable fields up to 37 T in a 34 mm bore. A broadband ^1^H-NMR probe was used enabling the in situ tuning of NMR frequencies between 800 MHz and 1.4 GHz. Data acquisition and analysis were performed by using a home-built variable-frequency NMR spectrometer covering Larmor frequency up to 2 GHz. The relaxation rates were measured at frequencies of 822 MHz/19.3 T, 1020 MHz/24.0 T, 1200 MHz/28.2 T, and 1382 MHz/32.5 T.

The sample temperature in the resistive magnet was maintained at 298 ± 2 K and measured by a thermometer located 1-2 cm above the sample. For temperature regulation, a nitrogen gas flowed through a heater element in a vacuum-isolated stainless steel tube of 16 mm diameter. The surrounding environment of the water-cooled resistive magnet operating between 10 and 20 MW caused some variations in the sample temperature that remained within the error limits (±2 K).

### 3.4. Extraction of Relaxivities $R_{1}$ and $R_{2}$

^1^H relaxation rates, $R_{1}$ and $R_{2}$, were measured for all concentrations *c* of a series with a dissolved Ln^3+^. Due to limited experimental time in the resistive magnet, we restricted ourselves to selected concentrations of 1, 5, and 10 mMol $L^{-1}$, as well as the pure solvent (c=0 mMol $L^{-1}$). $R_{1}$ values were obtained from fits of the inversion or saturation intensities $M_{z}\left(t_{\mathrm{rec}}\right)$ using

$$M_{z}\left(t_{\mathrm{rec}}\right)=M_{eq}\left(1-C\;\exp\left(-R_{1}\;t_{\mathrm{rec}}\right)\right),$$

where $t_{\mathrm{rec}}$ is the variable recovery time. The fit parameters are the equilibrium magnetization, $M_{eq}$, the inversion or saturation degree, *C*, and the $R_{1}$.

$R_{2}$ were obtained from fits of the echo maxima, $M_{⊥}\left(\tau_{i}\right)$, occurring at $\tau_{i}$ using

$$M_{⊥}\left(\tau_{i}\right)=M_{⊥}\left(0\right)\;\exp\left(-R_{2}\;\tau_{i}\right).$$

**Figure 9:**
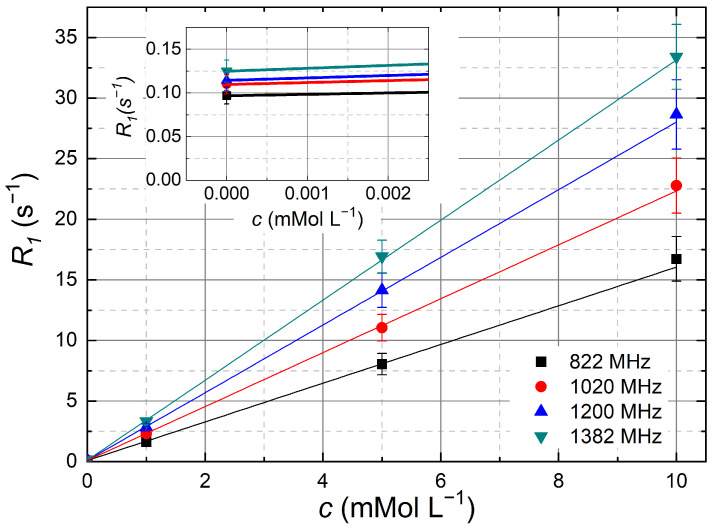
Concentration dependence of the longitudinal ^1^H relaxation rates $R_{1}$ of water with dissolved ErCl_3_ at various $f_{0}$ measured in the resistive magnet as defined in Equation (3). The slopes of the corresponding linear fits provide the relaxivities $r_{1}$. The inset shows the offset, which is the relaxation rate of the solvent $R_{1,\mathrm{solvent}}\;\approx$ 0.11 $s^{-1}$.

The fit parameters are the initial echo intensity at $M_{⊥}\left(0\right)$, and the $R_{2}$. As expected for homogeneous solutions, the longitudinal magnetization recovery curves were always found to be mono-exponential as well as the transverse magnetization decays, except for the cases where the field fluctuation of the resistive magnet perturbed the $R_{2}$ experiment. The relaxation rates $R_{i}$ at each field were plotted as a function of concentration *c* (Figure 9). The $R_{1}\left(c\right)$ of the Er^3+^ compound at various frequencies measured in the resistive magnet exemplarily shows the expected linearity. The relaxivities $r_{i}$ were extracted by fitting the following linear relation to the data

(3) $$R_{i}=r_{i}.c+R_{i,\mathrm{solvent}},$$

where $R_{i,\mathrm{solvent}}$, the relaxation rate of the pure solvent (9:1 D_2_O:H_2_O) is the offset in Figure 9 at *c* = 0 mMol $L^{-1}$. This value was found to be almost constant at $R_{1,\mathrm{solvent}}$ = 0.11 $s^{-1}$ over the entire frequency range from 20 to 1382 MHz with a variation of 0.05 $s^{-1}$. No deviation from linearity was observed for the slope, nor were any offsets other than $R_{i,\mathrm{solvent}}$ observed in any field, which indicates the absence of saturation effects, clustering, or errors in the concentration.

## 4. Conclusions

In summary, longitudinal and transverse ^1^H NMRD studies were performed over a wide Larmor frequency range (20 MHz up to 1382 MHz) for aqueous solutions of LnCl_3_ salts with Ln ∈ {Gd,Dy,Ho,Er}. Special attention was given to the quality and reliability of the NMRD data above 800 MHz in the resistive magnet, which faces challenges in field homogeneity and stability. We analyzed and validated our methodological NMRD approach to overcome these drawbacks: sufficient field homogeneity of 15 ppm can be obtained for sample sizes of 1 mm^3^. For the handling of the corresponding μL sample volumes, we developed special tools and methods. Time variations of the magnetic field in the resistive magnet have different impacts on the longitudinal and transverse NMRD experiments. We identified the sensitivity of the inversion pulse on the field fluctuations as the limiting factor for the relaxation experiments. This can be generally overcome for longitudinal relaxation by the progressive saturation recovery sequence. For transverse relaxation rates below a threshold given by the rate of the field fluctuations, no reliable data can be obtained. Whereas the use of stronger pulses and higher concentrations is a workaround for special cases, this restriction can only be lifted by the suppression of magnetic field fluctuations. This requires the development of an active field correction system. In the next phase, we validated the reliability of our NMRD studies in resistive magnets by comparative experiments with superconducting magnets at 800 MHz and 950 MHz. Precise temperature measurement and control were identified as crucial factors. These findings offer quantitative insights into the quality of NMRD studies in such magnets and their implications for future studies. For example, efficient temperature control would enable NMRD experiments at different temperatures for PRE studies where this is of interest.

Our experimental longitudinal NMRD results below 600 MHz are fully consistent with previous studies. The transverse NMRD was found to scale with the longitudinal one with a factor close to one that slightly increases with field, which is consistent with the current model. We modeled our longitudinal and transverse NMRD data for Dy^3+^, Er^3+^ and Ho^3+^ up to a frequency more than two times larger via the Solomon–Bloembergen–Morgan theory for inner sphere relaxation, plus an additional outer sphere contribution based on the Hwang–Freed theory. The model parameters are in agreement with previously published values and should enable the refinement of the existing microscopic model for NMRD induced by Ln^3+^ ions in aqueous solutions.

## Figures and Tables

**Table 1:** Table of fitted and fixed parameters used for NMRD modeling of Dy^3+^, Ho^3+^, and Er^3+^ relaxivity data. The electronic correlation time $\tau_{S}$ and rotational correlation time $\tau_{R}$ are the parameters fitted from the relaxivity data, and the values in brackets are the results of previous PRE studies [19]. The parameters that were fixed and their corresponding values are the correlation time of chemical exchange $\tau_{M}$, the number of coordinated water molecules in the inner sphere *q*, as well as the inner and outer sphere radii.

Ln^3+^	$\tau_{S}$ [ps]	$\tau_{R}$ [ps]	$\tau_{M}$ [ns]	*q*	$r_{\mathrm{is}}$ [Å]	$r_{\mathrm{os}}$ [Å]
Dy^3+^	0.3 [0.39]	50 [63]	2.6	8	3.1	4.2
Ho^3+^	0.21 [0.27]	45 [65]	5.2	8	3.1	4.2
Er^3+^	0.28 [0.31]	54 [61]	8.5	8	3.1	4.2

## Data Availability

Experimental datasets and modeling curves are available on request from the authors (R.N.D. or S.K.).

## Appendix A. Parameters of NMR Spectrometers

**Table A1:** Magnetic fields, magnet types, and experimental NMR parameters used in the ^1^H NMRD study on aqueous solutions of Ln^3+^ ions.

Facility	KIT					IBS	BRUKER	IBS	LNCMI
Frequency [MHz]	20	80	200	300	400	600	800	950	822–1382
Magnetic field [T]	0.47	1.9	4.7	7.0	9.4	14.1	18.8	22.3	19.3–32.5
Type of Magnet	Permanent		Superconducting						Resistive
Spectrometer type	Bruker	Bruker	Bruker						Home-made
	the minispec	Fourier	AVANCE						
Sample tube OD [mm]	5				1.7				1.2
Spectrometer	Bruker	Bruker							LabVIEW
software	the minispec	TOPSPIN 3+4							2013
Processing software	Origin 2023 (V. 10.0)	Bruker Dynamic Center Center (V.2.2)				Python (V.3.13.0)	Bruker Dynamic Center (V.2.2)	Python (V.3.13.0)	Matlab R2019b
$T_{1}$ parameters									
$T_{1}$ sequence	PSR	IR							PSR
No. of scans	4								1
Exp. dimension	2D								
No. of recovery delays	16								20–30
$T_{2}$ parameters									
$T_{2}$ sequence	CPMG: ${\left(\pi /2\right)}_{y}-{\left(\Delta_{e}-\pi_{x}-\Delta_{e}\right)}_{2i}$								
No. of scans	4					8			1
Exp. dimension	1D	2D							1D
No. of echos	$2\;i_{\max}$ = 100	list with 16 or 32 elements, $2\;i_{\max}$ = 500							$2\;i_{\max}$ > 500

KIT: Karlsruhe Institute of Technology, Karlsruhe, Germany; IBS: Institut de Biologie Structurale, Grenoble, France; BRUKER: Bruker BioSpin, Ettlingen, Germany; LNCMI: Laboratoire National des Champs Magnétiques Intenses, Grenoble, France; OD: outer diameter; PSR: Progressive saturation recovery pulse sequence; IR: Inversion recovery pulse sequence; CPMG: Carr–Purcell–Meiboom–Gill pulse sequence

## Appendix B. Parameters of the CPMG Experiments

**Table A2:** Nutation frequencies $f_{1}$, pulse durations for π/2 and π pulses and durations of the interpulse delay $\Delta_{e}$ of the CPMG sequence, given by ${\left(\pi /2\right)}_{y}-{\left(\Delta_{e}-\pi_{x}-\Delta_{e}\right)}_{2i}$. For the resistive magnet (last 4 lines), the table also shows the upper limits for $f_{\mathrm{off}}/f_{1}$ and $\Delta_{e}\;f_{\mathrm{off}}$ using our maximum off-resonance frequency $f_{\mathrm{off}}$ = 60 kHz, since they were used in [31] to quantify systematic errors induced by off-resonant effects. For superconducting magnets, the pulses were always on-resonant (spin lock) and no effects of field fluctuations of $R_{2}$ were observed in any measurement.

$f_{0}$	$f_{1}$	π/2	π	$\Delta_{e}$	$f_{\mathrm{off}}$	$f_{\mathrm{off}}/f_{1}$	$\Delta_{e}\;f_{\mathrm{off}}$
[MHz]	[kHz]	[μs]	[μs]	[μs]	[kHz]		
20	31	8	16	variable	n/a	n/a	n/a
80	22	11.3	22.6	variable	n/a	n/a	n/a
200	58	4.3	8.6	variable	n/a	n/a	n/a
300	18	14	28	variable	n/a	n/a	n/a
400	25	10	20	variable	n/a	n/a	n/a
600	28	9	18	variable	n/a	n/a	n/a
800	40	6.3	12.6	variable	n/a	n/a	n/a
950	28	9	18	variable	n/a	n/a	n/a
822	109	2.3	4.6	66	60	0.5	3.96
1020	100	2.5	5	66	60	0.6	3.96
1200	104	2.4	4.8	66	60	0.6	3.96
1382	69	3.6	7.2	66	60	0.9	3.96

Variable: $\Delta_{e}\approx 5\;R_{2}^{-1}/\left(4\;i_{\max}\right)$, i.e., the last echo time corresponded to the expected $5\;T_{2}=5\;R_{2}^{-1}$ that was calculated by inter- and extrapolation of the already recorded $R_{2}$. In the case of deviations, the experiment was repeated with an optimized $\Delta_{e}$. Further details can be found in [39].

## Appendix C. Properties of Lanthanide Ions

**Table A3:** Magnetic, structural and dynamic properties of Ln^3+^ ions in aqueous solutions used in this NMRD study. The last column of the table lists the references from which the parameters are taken.

Compounds	Ln^3+^	Gd^3+^	Dy^3+^	Ho^3+^	Er^3+^	Ref.
Magnetic Properties	*S*	7/2	5/2	2	3/2	
	*L*	0	5	6	6	
	*J*	7/2	15/2	8	15/2	
	*g*	7/2	4/3	5/4	6/5	
	$\mu_{\mathrm{eff}}$ [$\mu_{B}$]	13.9	10.6	10.6	9.6	
	Anisotropy	none	oblate	oblate	prolate	[43]
Coordination no.	*q*	9	8	8	8	[19]
Exchange time	$\tau_{M}$ [$10^{-9}$ s]	0.9	2.6	5.2	8.5	[32]
Inner-sphere	$r_{is}$ [$10^{-10}$ m]	≈3.1	≈3.1	≈3.1	≈3.1	[18,19]
radius						
Outer-sphere	$r_{os}$ [$10^{-10}$ m]	≈4.2	≈4.2	≈4.2	≈4.2	[14]
radius						

$\mu_{\mathrm{eff}}=g_{j}\sqrt{J(J+1)}\mu_{B}$; Coordination number *q*: Number of coordinated water molecules in the first coordination sphere.

## Appendix D. PRE Formulas for the Modeling of the NMRD Profiles

In this part, we briefly resume the PRE model used for the modeling of our NMRD data. The equations were taken from [3,11,20,36]. The relaxation rate $R_{i}$ is the sum of inner sphere relaxation, $R_{i,is}$ and outer sphere relaxation $R_{i,os}$

$$R_{i}=R_{i,is}+R_{i,os}.$$

The inner sphere longitudinal relaxation is given by

$$R_{1,is}=fq\frac{1}{T_{1,M}+\tau_{M}}.$$

It depends on the ratio of the concentration of the PRE compound and the solvent, *f*; the number of water molecules in the first coordination sphere of the complex, *q*; the chemical exchange time of the bound water molecule with the free solvent, $\tau_{M}$; and the relaxation rate of the bound nuclei $R_{1,M}=1/T_{1,M}$, which is the sum of the dipolar $R_{1,is}^{DD}$ and Curie spin $R_{1,is}^{C}S$ contributions

$$R_{1,M}=R_{1,is}^{DD}+R_{1,is}^{CS}.$$

The dipolar contribution for $\omega_{S}\gg \omega_{I}$ is

$$R_{1,is}^{DD}=\frac{2}{15}{\left(\frac{\mu_{0}}{4\pi }\right)}^{2}\gamma_{I}^{2}\overset{\mu_{\mathrm{eff}}^{2}}{\overset{︷}{\mu_{B}^{2}g_{j}^{2}J\left(J+1\right)}}\frac{1}{r^{6}}\left[3j\left(\omega_{I},\tau_{c1}\right)+7j\left(\omega_{S},\tau_{c2}\right)\right],$$

where $\gamma_{I}$ is the nuclear gyromagnetic ratio, $\mu_{B}$ is the Bohr magneton, $g_{j}$ is the Landé factor, and *J* is the total electronic spin. The spectral density $j(\omega ,\tau_{c})$ is given by

$$j\left(\omega ,\tau_{c}\right)=\frac{\tau_{c}}{1+\omega^{2}\tau_{c}^{2}}.$$

$\tau_{c1,2}$ is the correlation time of the inner sphere dipolar coupling

$$\tau_{c1,2}^{-1}=\tau_{R}^{-1}+\tau_{M}^{-1}+\tau_{S1,2}^{-1},$$

where $\tau_{R}$ is the rotational correlation time and $\tau_{S1,2}$ are the longitudinal and transverse electronic relaxation times.

The Curie spin contribution is

$$R_{1,is}^{CS}=\frac{2}{5}{\left(\frac{\mu_{0}}{4\pi }\right)}^{2}\gamma_{I}^{2}\overset{\mu_{\mathrm{eff}}^{4}}{\overset{︷}{\mu_{B}^{4}g_{j}^{4}J^{2}{\left(J+1\right)}^{2}}}\frac{1}{r^{6}}\frac{B_{0}^{2}}{{\left(3k_{B}T\right)}^{2}}3j\left(\omega_{I},\tau_{cs}\right),$$

with the Curie spin correlation time

$$\tau_{cs}^{-1}=\tau_{R}^{-1}+\tau_{M}^{-1}$$

that only depends on $\tau_{R}$ and $\tau_{M}$.

$B_{0}=f_{0}/\gamma_{I}$ is the applied magnetic field, $k_{B}$ is the Boltzmann constant, and *T* is the temperature.

The outer sphere relaxation rate is the sum of dipolar ($R_{1,os}^{DD}$) and Curie ($R_{1,os}^{CS}$) contributions, which are given by

$$\begin{array}{ccc} R_{1,os}^{DD} & = & \frac{16\pi }{135}{\left(\frac{\mu_{0}}{4\pi }\right)}^{2}\gamma_{I}^{2}\mu_{B}^{2}g_{J}^{2}N_{A}c\frac{1}{aD}\left\{6\left[J\left(J+1\right)-S_{C}\coth\frac{\chi }{2J}-S_{C}^{2}\right]j_{o}\left(\omega_{I},\tau_{D},\tau_{S}\right)\right.\\ & & \left.+7\coth\frac{\chi }{2J}\cdot S_{C}\cdot j_{O}\left(\omega_{S},\tau_{D},\tau_{S}\right)\right\}\;\mathrm{and}\\ R_{1,os}^{CS} & = & \frac{32\pi }{405}{\left(\frac{\mu_{0}}{4\pi }\right)}^{2}\gamma_{I}^{2}\mu_{B}^{2}g_{J}^{2}N_{A}c\frac{1}{aD}S_{C}^{2}j^{A}\left(\omega_{I}\tau_{D}\right). \end{array}$$

$N_{A}$ is Avogadro’s constant, c is the molar concentration of the PM compound, $a=r_{os}$ is the distance of closest approach, D is the relative diffusion constant, $\tau_{D}=a^{2}/D$ is the translational correlation time and $S_{C}$ is the time-averaged Curie spin

$$S_{C}=\langle S_{z}\rangle =J\left(\frac{2J+1}{2J}\coth\frac{(2J+1)\chi }{2J}-\frac{1}{2J}\coth\frac{\chi }{2J}\right),$$

where χ is given by

$$\chi =\frac{JB_{0}\mu_{B}g_{J}}{kT}.$$

The spectral density function for the dipolar contribution, $j_{o}$, depends on $\tau_{D}$ and $\tau_{S}$

jo(ω,τD,τS)=Re1+Ω1/2/41+Ω1/2+4Ω/9+Ω3/2/9withΩ=(iω+1/τS)τD,

whereas the one for the Curie contribution,$j^{A}$, only depends on $\tau_{D}$

jA(ω,τD)=Re1+Ω1/2/41+Ω1/2+4Ω/9+Ω3/2/9withΩ=iωτD.

In a similar way, the ^1^H transverse relaxation, $R_{2}$, can be decomposed into inner and outer sphere contributions. The inner sphere contribution, $R_{2,is}$, is related to the transverse relaxation rate $R_{2M}=1/T_{2M}$ of the coordinated water molecule

$$R_{2,is}=fq\frac{1}{\tau_{M}}\frac{\frac{1}{T_{2M}^{2}}+\frac{1}{\tau_{M}T_{2M}}+\Delta \omega_{M}^{2}}{{\left(\frac{1}{T_{2M}}+\frac{1}{\tau_{M}}\right)}^{2}+\Delta \omega_{M}^{2}},$$

where $\Delta \omega_{M}$ is the chemical shift term, given by the sum of contact and pseudocontact terms. It is proportional to the external magnetic field.

$R_{2M}=1/T_{2M}$ contains dipolar ($R_{2}^{DD}$), dipolar Curie ($R_{2}^{CS}$) and contact Curie ($R_{2}^{CC}$) contributions, which are absent for the case of Ln^3+^ ions [44]. $R_{2}^{DD}$ and $R_{2}^{CS}$ are given by

$$\begin{array}{ccc} R_{2,is}^{DD} & = & \frac{1}{15}{\left(\frac{\mu_{0}}{4\pi }\right)}^{2}\gamma_{I}^{2}\overset{\mu_{\mathrm{eff}}^{2}}{\overset{︷}{\mu_{B}^{2}g_{j}^{2}J\left(J+1\right)}}\frac{1}{r^{6}}\left[4\tau_{C1}+3j\left(\omega_{I},\tau_{c1}\right)+13j\left(\omega_{S},\tau_{c2}\right)\right]\;\mathrm{and}\\ R_{2,is}^{CS} & = & \frac{1}{5}{\left(\frac{\mu_{0}}{4\pi }\right)}^{2}\gamma_{I}^{2}\overset{\mu_{\mathrm{eff}}^{4}}{\overset{︷}{\mu_{B}^{4}g_{j}^{4}J^{2}{\left(J+1\right)}^{2}}}\frac{1}{r^{6}}\frac{B_{0}^{2}}{{\left(3k_{B}T\right)}^{2}}\left[4\tau_{cs}+3j\left(\omega_{I},\tau_{cs}\right)\right]. \end{array}$$

The outer sphere contributions for the dipolar and Curie relaxation are

R2,osDD=16π135μ04π2γI2μB2gJ2NAc1aDJ(J+1)−SCcothχ2J−SC2  3jo(ωI,τD,τS)+4jo(0,τD,τS)+6.5cothχ2J·SC·jo(ωS,τD,τS)andR2,osCS=16π405μ04π2γI2μB2gJ2NAc1aDSC23jA(ωI,τD)+4jA(0,τD).

The relevant properties for NMRD studies of the investigated Ln^3+^ ions in aqueous solutions are summarized in Table A3. The inner sphere radius $r_{is}$ and the number of the coordinated water molecules *q* are adapted from previous studies [14,19,32].

## Appendix E. Experimental Relaxivity Data

**Table A4:** Table of experimental longitudinal relaxivities $r_{1}$.

$f_{0}$	$r_{1}$(Gd)	$r_{1}$(Dy)	$r_{1}$(Ho)	$r_{1}$(Er)
[MHz]	[$s^{-1}$mMol^−1^L]			
20	14.1 ± 0.4	0.520 ± 0.01	0.360 ± 0.009	0.416 ± 0.01
80	13.5 ± 0.3	0.560 ± 0.01	0.384 ± 0.010	0.403 ± 0.01
200	15.1 ± 0.4	0.652 ± 0.02	0.494 ± 0.01	0.470 ± 0.01
300	13.7 ± 0.3	0.760 ± 0.02	0.610 ± 0.02	0.556 ± 0.01
400	13.8 ± 0.7	0.973 ± 0.05	0.816 ± 0.04	0.685 ± 0.03
600	13.0 ± 0.3	1.40 ± 0.04	1.28 ± 0.03	0.999 ± 0.02
800	13.2 ± 0.3	2.08 ± 0.05	1.98 ± 0.05	1.50 ± 0.04
950	12.1 ± 0.3	2.48 ± 0.06	2.37 ± 0.06	1.80 ± 0.04
822	13.5 ± 0.7	2.40 ± 0.1	2.26 ± 0.1	1.55 ± 0.08
1020	13.7 ± 0.7	3.19 ± 0.2	3.08 ± 0.2	2.23 ± 0.1
1200	13.7 ± 0.7	4.14 ± 0.2	3.93 ± 0.2	2.86 ± 0.1
1382	13.3 ± 0.7	4.44 ± 0.2	4.52 ± 0.2	3.28 ± 0.2

**Table A5:** Table of experimental transverse relaxivities $r_{2}$.

$f_{0}$	$r_{2}$(Gd)	$r_{2}$(Dy)	$r_{2}$(Ho)	$r_{2}$(Er)
[MHz]	[$s^{-1}$mMol^−1^L]			
20	17.0 ± 0.8	0.574 ± 0.03	0.390 ± 0.02	0.442 ± 0.02
80	15.8 ± 0.8	0.550 ± 0.03	0.385 ± 0.02	0.396 ± 0.02
200	17.9 ± 0.9	0.688 ± 0.03	0.530 ± 0.03	0.490 ± 0.02
300	16.1 ± 0.8	0.840 ± 0.04	0.687 ± 0.03	0.594 ± 0.03
400	18.1 ± 0.9	1.07 ± 0.05	0.915 ± 0.05	0.789 ± 0.04
600	15.7 ± 0.8	1.70 ± 0.08	1.48 ± 0.07	1.15 ± 0.06
800	16.3 ± 0.8	2.49 ± 0.1	2.37 ± 0.1	1.76 ± 0.09
950	14.8 ± 0.7	3.01 ± 0.2	2.94 ± 0.1	2.21 ± 0.1
822	18.2 ± 1	6.11 ± 1	3.86 ± 0.3	3.56 ± 0.5
1020	18.0 ± 1	4.71 ± 0.3	4.46 ± 0.5	3.76 ± 0.8
1200	18.4 ± 1	6.11 ± 1	5.79 ± 0.5	3.95 ± 0.4
1382	18.2 ± 1	6.81 ± 0.8	7.15 ± 0.6	4.92 ± 0.6
